# COVID-19 vaccine effectiveness against symptomatic infection with SARS-CoV-2 BA.1/BA.2 lineages among adults and adolescents in a multicentre primary care study, Europe, December 2021 to June 2022

**DOI:** 10.2807/1560-7917.ES.2024.29.13.2300403

**Published:** 2024-03-28

**Authors:** Charlotte Lanièce Delaunay, Iván Martínez-Baz, Noémie Sève, Lisa Domegan, Clara Mazagatos, Silke Buda, Adam Meijer, Irina Kislaya, Catalina Pascu, AnnaSara Carnahan, Beatrix Oroszi, Maja Ilić, Marine Maurel, Aryse Melo, Virginia Sandonis Martín, Camino Trobajo-Sanmartín, Vincent Enouf, Adele McKenna, Gloria Pérez-Gimeno, Luise Goerlitz, Marit de Lange, Ana Paula Rodrigues, Mihaela Lazar, Neus Latorre-Margalef, Gergő Túri, Jesús Castilla, Alessandra Falchi, Charlene Bennett, Virtudes Gallardo, Ralf Dürrwald, Dirk Eggink, Raquel Guiomar, Rodica Popescu, Maximilian Riess, Judit Krisztina Horváth, Itziar Casado, Mª del Carmen García, Mariëtte Hooiveld, Ausenda Machado, Sabrina Bacci, Marlena Kaczmarek, Esther Kissling, on behalf of the European Primary Care Vaccine Effectiveness Group

**Affiliations:** 1 Epiconcept, Paris, France; 2 Instituto de Salud Pública de Navarra – IdiSNA, Pamplona, Spain; 3 Consortium for Biomedical Research in Epidemiology and Public Health (CIBERESP), Madrid, Spain; 4 Sorbonne Université, INSERM, Institut Pierre Louis d'épidémiologie et de Santé Publique (IPLESP UMRS 1136), Paris, France; 5 Health Protection Surveillance Centre, Dublin, Ireland; 6 National Centre of Epidemiology, CIBERESP, Carlos III Health Institute, Madrid, Spain; 7 Department for Infectious Disease Epidemiology, Respiratory Infections Unit, Robert Koch Institute, Berlin, Germany; 8 National Institute for Public Health and the Environment (RIVM), Bilthoven, the Netherlands; 9 Instituto Nacional de Saúde Dr. Ricardo Jorge, Lisbon, Portugal; 10 Cantacuzino National Military Medical Institute for Research and Development, Bucharest, Romania; 11 Public Health Agency of Sweden, Stockholm, Sweden; 12 National Laboratory for Health Security, Epidemiology and Surveillance Centre, Semmelweis University, Budapest, Hungary; 13 Croatian Institute of Public Health (CIPH), Zagreb, Croatia; 14 National Centre for Microbiology, Carlos III Health Institute, Madrid, Spain; 15 Institut Pasteur, Pasteur International Bioresources network (PIBnet), Plateforme de Microbiologie Mutualisée (P2M), Paris, France; 16 Institut Pasteur, Centre National de Référence Virus des Infections Respiratoires (CNR VIR), Paris, France; 17 Laboratoire de Virologie, Université de Corse-Inserm, Corte, France; 18 National Virus Reference Laboratory, University College Dublin, Dublin, Ireland; 19 Dirección General de Salud Pública y Ordenación Farmacéutica, Junta de Andalucía, Sevilla, Spain; 20 National Reference Centre for Influenza, Robert Koch Institute, Berlin, Germany; 21 National Institute of Public Health, Bucharest, Romania; 22 Subdirección de Epidemiología, Dirección General de Salud Pública, Servicio Extremeño de Salud, Mérida, Spain; 23 Nivel, Utrecht, the Netherlands; 24 European Centre for Disease Prevention and Control, Stockholm, Sweden; 25 The members of the group are listed under Acknowledgements

**Keywords:** COVID-19, SARS-CoV-2, BA.1, BA.2, vaccine effectiveness, symptomatic infection, test-negative design

## Abstract

**Background:**

Scarce European data in early 2021 suggested lower vaccine effectiveness (VE) against SARS-CoV-2 Omicron lineages than previous variants.

**Aim:**

We aimed to estimate primary series (PS) and first booster VE against symptomatic BA.1/BA.2 infection and investigate potential biases.

**Methods:**

This European test-negative multicentre study tested primary care patients with acute respiratory symptoms for SARS-CoV-2 in the BA.1/BA.2-dominant period. We estimated PS and booster VE among adults and adolescents (PS only) for all products combined and for Comirnaty alone, by time since vaccination, age and chronic condition. We investigated potential bias due to correlation between COVID-19 and influenza vaccination and explored effect modification and confounding by prior SARS-CoV-2 infection.

**Results:**

Among adults, PS VE was 37% (95% CI: 24–47%) overall and 60% (95% CI: 44–72%), 43% (95% CI: 26–55%) and 29% (95% CI: 13–43%) < 90, 90–179 and ≥ 180 days post vaccination, respectively. Booster VE was 42% (95% CI: 32–51%) overall and 56% (95% CI: 47–64%), 22% (95% CI: 2–38%) and 3% (95% CI: −78% to 48%), respectively. Primary series VE was similar among adolescents. Restricting analyses to Comirnaty had little impact. Vaccine effectiveness was higher among older adults. There was no signal of bias due to correlation between COVID-19 and influenza vaccination. Confounding by previous infection was low, but sample size precluded definite assessment of effect modification.

**Conclusion:**

Primary series and booster VE against symptomatic infection with BA.1/BA.2 ranged from 37% to 42%, with similar waning post vaccination. Comprehensive data on previous SARS-CoV-2 infection would help disentangle vaccine- and infection-induced immunity.

**Key public health message**

**What did you want to address in this study and why?**

COVID-19 vaccine effectiveness can vary across populations and periods depending on multiple factors including the SARS-CoV-2 variants circulating, the time since last vaccination and the proportion of people who have been recently infected. We estimated the effectiveness of primary series and first booster vaccination against symptomatic infection with Omicron lineages BA.1 and BA.2 in a European study in 10 countries, up to June 2022.

**What have we learnt from this study?**

The overall effectiveness of primary series vaccination and booster vaccination was around 40%, meaning that the risk of symptomatic COVID-19 was 40% lower among vaccinated people than among unvaccinated people. Vaccines provided lower protection against symptomatic infection with BA.1 and BA.2 than with previously circulating variants (e.g. Delta), and this protection decreased with time.

**What are the implications of your findings for public health?**

Our results suggest that the timing of COVID-19 vaccination is key: vaccines should be administered in the weeks preceding periods of high SARS-CoV-2 circulation, and adapted vaccines could be considered. Primary series and booster vaccination had similar effectiveness, therefore if may be sufficient to estimate VE by time since last dose among those who received at least primary series vaccination, rather than by number of doses.

## Background

As of March 2023, the European Medicines Agency (EMA) authorised seven COVID-19 vaccines for adults: Comirnaty (Pfizer/BioNTech), Spikevax (Moderna), Vaxzevria (AstraZeneca), Jcovden (Janssen), Nuvaxovid (Novavax), Valneva (Valneva) and VidPrevtyn Beta (Sanofi Pasteur) [1]. These vaccines were first developed against the original strain of severe acute respiratory syndrome coronavirus 2 (SARS-CoV-2), except for VidPrevtyn that targets the Beta variant. In autumn 2022, the EMA additionally authorised bivalent Comirnaty and Spikevax mRNA vaccines developed based on both the original strain and the BA.1 and BA.4/BA.5 Omicron lineages, however, this was after the date period covered in our current study. The minimum age for receiving Comirnaty and Spikevax has decreased, with adolescents and children aged ≥ 6 months now eligible to receive monovalent and bivalent mRNA vaccines [1]. Adolescents (aged ≥ 12 years) can also receive Nuvaxovid.

Post-marketing studies are essential to monitor vaccine effectiveness (VE). The test-negative design (TND) is widely used to estimate influenza VE, and many TND studies have been adapted to assess COVID-19 VE [2]. This approach can minimise biases due to healthcare-seeking behaviour and case misclassification, provided that the test-negative controls are representative of the source population from which cases arise in terms of vaccination [3,4]. Generating robust COVID-19 VE estimates is challenging due to emerging variants and diverse vaccine products, vaccination schedules and patterns of exposure to SARS-CoV-2. In this context, it is critical to leverage information collected in COVID-19 VE studies to investigate and quantify potential biases.

In TND studies, positive correlation between influenza vaccination and COVID-19 vaccination can lead to confounding and bias VE downwards for both viruses [5-8]. For example, in a TND, SARS-CoV-2 controls include SARS-CoV-2-negative, influenza-positive patients. These patients are less likely to be vaccinated against influenza and, assuming correlation between influenza and COVID-19 vaccination, are also less likely to be vaccinated against COVID-19. In this case, exclusion of influenza-positive, SARS-CoV-2-negative controls from COVID-19 VE analyses can provide unbiased estimates [5].

Confounding by history of SARS-CoV-2 infection may also arise. Previous infection may impact the probability of receiving a COVID-19 vaccine (e.g. if the perception of risks associated with a new SARS-CoV-2 infection differs by previous infection status) and the risk of infection (due to immunity conferred by past exposure to SARS-CoV-2) [9]. As vaccine-conferred immunity wanes, patients initially protected by vaccination may have higher odds of COVID-19 in subsequent waves than unvaccinated individuals immunised by natural infection, resulting in apparent loss of vaccine benefit [10]. Finally, there could be effect modification by previous infection status, as vaccination may provide greater absolute benefit to people without pre-existing immunity [9,11].

COVID-19 VE was high against symptomatic infection with the original SARS-CoV-2 strain and the Alpha, Beta and Delta variants [2,12-14]. The Omicron variant started circulating in the winter 2021/22 in Europe. Results from a global systematic review and meta-analysis suggest lower VE against symptomatic infection with Omicron lineages BA.1 and BA.2, with rapid waning of immunity [15]. In that meta-analysis, data were sparse for VE > 6 months post vaccination, and few European studies were included [15]. We need robust pan-European estimates of VE against Omicron infections for all age groups and time since vaccination intervals, to inform COVID-19 vaccination strategies in the context of this variant.

VEBIS (Vaccine Effectiveness, Burden and Impact Studies) is a multicentre TND study conducted among primary care patients in European Union (EU)/European Economic Area (EEA) countries. We estimated original-strain COVID-19 VE against symptomatic infection with BA.1/BA.2 at primary care level of full primary series (PS) and PS plus first booster dose among adults, and PS among adolescents. We also investigated potential sources of bias in COVID-19 VE estimates in this setting.

## Methods

### Study design and population

We included 10 European sites in the VEBIS primary care study: France (FR), Germany (DE), Hungary (HU), Ireland (IE), the Netherlands (NL), Portugal (PT), Romania (RO), Spain, national (ES), Spain, Navarra region (NA) and Sweden (SE). All study sites have experience from influenza VE studies before the COVID-19 pandemic using the same study design through the I-MOVE multicentre network. Many have also participated in COVID-19 VE studies since 2021 (through the I-MOVE-COVID-19 network) [2,16-18].

In each site, participating physicians swab all or a systematic sample of patients consulting with symptoms meeting the EU acute respiratory infection (ARI) definition (sudden onset of symptoms AND at least one respiratory symptom (cough, sore throat, shortness of breath, coryza) AND a clinician’s judgement that the illness is due to an infection) [19]. Site-specific variations in case definition are described in the Supplement.

**Figure 1:**
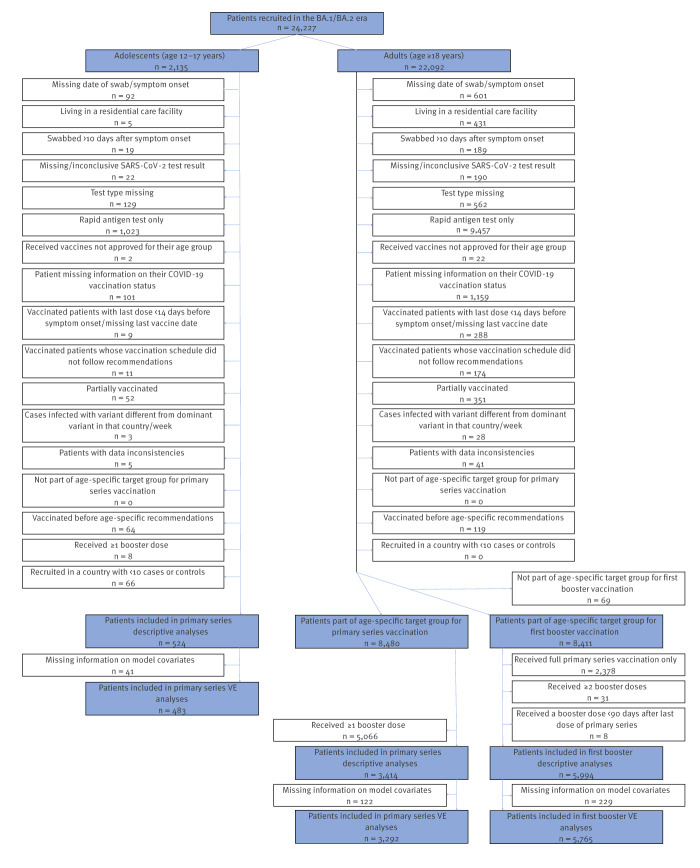
Study eligibility flowchart, VEBIS primary care study on COVID-19 vaccine effectiveness, EU/EEA, December 2021–June 2022 (n = 24,227)

EEA: European Economic Area; EU: European Union; SARS-CoV-2: severe acute respiratory syndrome coronavirus 2; VE: vaccine effectiveness; VEBIS: Vaccine Effectiveness, Burden and Impact Studies.

Demographic and clinical information is collected via an interview and/or via linkage to electronic medical records. Information on COVID-19 vaccination is self-reported or retrieved from national vaccination registries. Cases and controls are patients testing RT-PCR-positive and RT-PCR-negative for SARS-CoV-2, respectively. Some sites also test biological samples of selected patients for influenza viruses and other respiratory pathogens. Study exclusion criteria are presented in Figure 1.

### Study period

Data included in this analysis were restricted to the BA.1/BA.2-dominant period (December 2021 to June 2022), defined as weeks in which BA.1 and/or BA.2 represented ≥ 90% of viruses sequenced in the country of interest, according to the Global Initiative on Sharing All Influenza Data (GISAID) or The European Surveillance System (TESSy) databases [20].

### Data management

In one site (NA), the data collected did not include symptom onset date. For this site, we therefore imputed all missing symptom onset dates as follows, using data from other sites:

Onset date = swab date − median number of days between onset date and swab date

Because reasons for missing data may have been different, patients from other sites who were missing a symptom onset date were excluded from our analyses (Figure 1). We excluded study sites with < 10 cases and/or < 10 controls. In NA, a comprehensive surveillance system was used rather than a sentinel system, yielding higher sample sizes than other sites. To avoid over-weighting this site, we randomly sampled 60% of NA patients (regardless of case or control status) per week of swab, before applying exclusion criteria. The 60% sampling was based on discussions among study partners around maximum acceptable contributions to the multicentre study and resulted in a contribution of 28–62% to the study, depending on the analysis.

### Vaccination definition

Patients included in VE analyses belonged to age-specific target groups recommended for COVID-19 vaccination or booster doses on their swab date. Patients who had not received any dose of COVID-19 vaccine and those vaccinated on the day of symptom onset were classified as unvaccinated; patients vaccinated 1–13 days before symptom onset were excluded from analyses. The PS consisted of one or two doses according to vaccine licensure. Patients who had received booster doses were excluded from PS VE analyses.

Patients were included in booster dose VE if they had received only one monovalent vaccine booster dose ≥ 90 days after completing the PS. Bivalent mRNA vaccines were not available during the study period.

### Descriptive analyses

Descriptive analyses were conducted separately among adults (i.e. age ≥ 18 years) and adolescents (i.e. age 12–17 years), and separately for populations included in PS and first booster analyses. We described the number of cases and controls by week of swab, the distribution of time between last vaccination and symptom onset (among vaccinated patients), and baseline characteristics of cases and controls.

### Vaccine effectiveness analyses

We generated all VE estimates using complete case analyses, dropping records with missing values for the variables included in the analysis. For both PS and booster VE estimates, we used unvaccinated patients as the reference group.

Among adults, we estimated PS and first booster VE by time since vaccination for all vaccine products and for those who received Comirnaty as PS vaccination (the most commonly administered brand), by age group and by presence of chronic condition. Among adolescents, we estimated PS VE by time since vaccination for all products and for those who received Comirnaty as PS vaccination; few adolescents had received a booster. We used logistic regression models to estimate VE, and all models were adjusted for the following potential confounders: study site, symptom onset date, age, sex, and presence of chronic condition (except in analyses stratified by chronic condition status). A description of functional forms (e.g. categorical, linear, quadratic, splines) explored for modelling variables is available in the Supplement. This modelling approach is hereafter referred to as ‘per protocol’.

### Methods for small sample sizes

To investigate small sample size bias, we duplicated VE analyses for which we had < 10 events per model parameter using Firth’s penalised regression approach [21]. If the per protocol and penalised regression estimates differed by ≥ 10%, we do not report that estimate [22]. Further, we do not report estimates for populations including < 20 vaccinated or unvaccinated individuals.

### Sensitivity analyses

Using data collected among adults, we investigated whether a potential correlation between COVID-19 vaccination and influenza vaccination could bias COVID-19 VE [5]. We restricted data to sites providing information on influenza RT-PCR testing. As recommended by Doll et al. [5], we compared the following estimates for PS and first booster VE (separately): (i) per protocol COVID-19 VE and (ii) COVID-19 VE obtained by excluding influenza-positive controls, using per-protocol modelling. We also evaluated the influence that the NA site had on our VE results by replicating the main analyses excluding patients recruited in NA. We conducted this sensitivity analysis among adults only, due to limited sample size for adolescents.

### Secondary analyses

We also explored potential effect modification or confounding by history of SARS-CoV-2 infection, selecting sites that collected this information. For effect modification, we separately estimated COVID-19 VE among patients who reported a previous infection with SARS-CoV-2, and among those who reported no previous infection. In addition, we estimated protection against SARS-CoV-2 among the following three groups, using the unvaccinated, never infected as a reference: (i) unvaccinated, previously infected, (ii) vaccinated, never infected and (iii) vaccinated, previously infected. For confounding, we estimated VE among people with information on previous SARS-CoV-2 infection, adjusting for previous infection, and compared this estimate with that obtained with per-protocol modelling in the same population.

## Results

### Descriptive analyses

**Figure 2:**
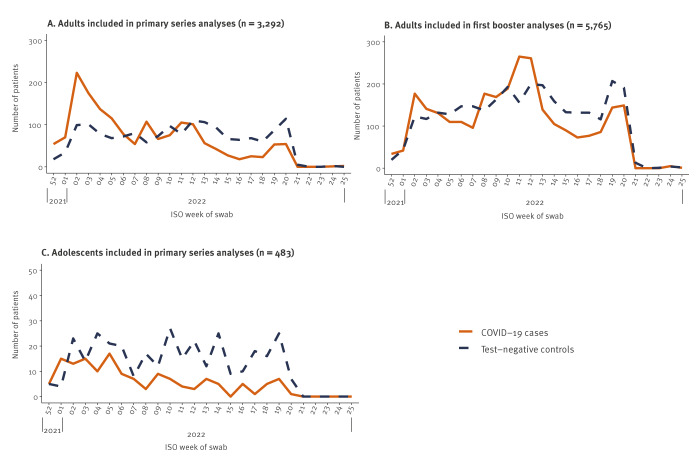
Recruited COVID-19 cases and test-negative controls, by week of swab, VEBIS primary care study, EU/EEA, December 2021–June 2022

EEA: European Economic Area; EU: European Union; ISO: International Organization for Standardization; VEBIS: Vaccine Effectiveness, Burden and Impact Studies.

We recruited a total of 24,227 patients from 10 study sites in the BA.1/BA.2 era (December 2021–June 2022; Figures 1 and 2). We included 3,292 adults and 483 adolescents in the PS analyses and 5,765 adults in the first booster analyses. We excluded 9,457 adults (6,514 from NA, 2,939 from FR, three from PT and one from ES) and 1,023 adolescents (646 from NA and 377 from FR) because they were tested with rapid antigen tests. These patients had been recruited with the same criteria as patients tested with RT-PCR.

**Figure 3:**
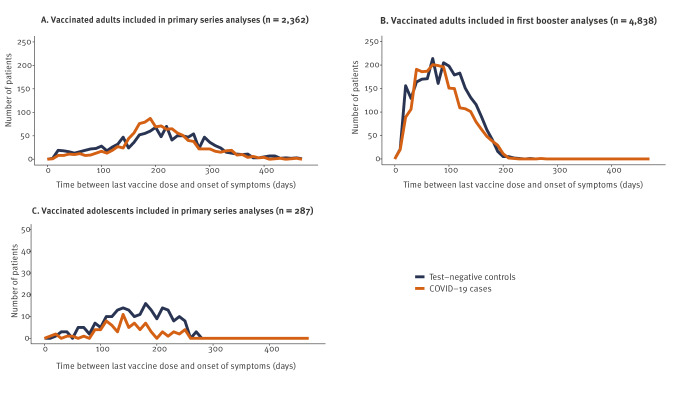
Vaccinated COVID-19 cases and test-negative controls, by time from last vaccine dose to symptom onset, VEBIS primary care study, EU/EEA, December 2021–June 2022

EEA: European Economic Area; EU: European Union; VEBIS: Vaccine Effectiveness, Burden and Impact Studies.

The median time from last vaccination to symptom onset among vaccinated patients was 205 days (interquartile range (IQR): 162–258), 87 days (IQR: 55–122) and 160 days (IQR: 122–202) among, respectively, adults included in PS analyses, adults included in first booster analyses and adolescents included in PS analyses (Figure 3).

Among adults included in PS analyses, 50% (838/1,661) of cases and 29% (468/1,631) of controls were recruited in a BA.1-dominant period (December 2021–February 2022), and 31% (510/1,661) of cases and 53% (865/1,631) of controls in a BA.2-dominant period (February–June 2022). Among adults included in first booster analyses, 27% (757/2,771) of cases and 24% (714/2,994) of controls were recruited in a BA.1-dominant period, and 51% (1,408/2,771) of cases and 56% (1,672/2,994) of controls in a BA.2-dominant period. Among adolescents included in PS analyses, 57% (84/148) of cases and 33% (112/335) of controls were recruited in a BA.1-dominant period, and 27% (40/148) of cases and 48% (161/335) of controls in a BA.2-dominant period.

### Vaccine effectiveness analyses

**Figure 4:**
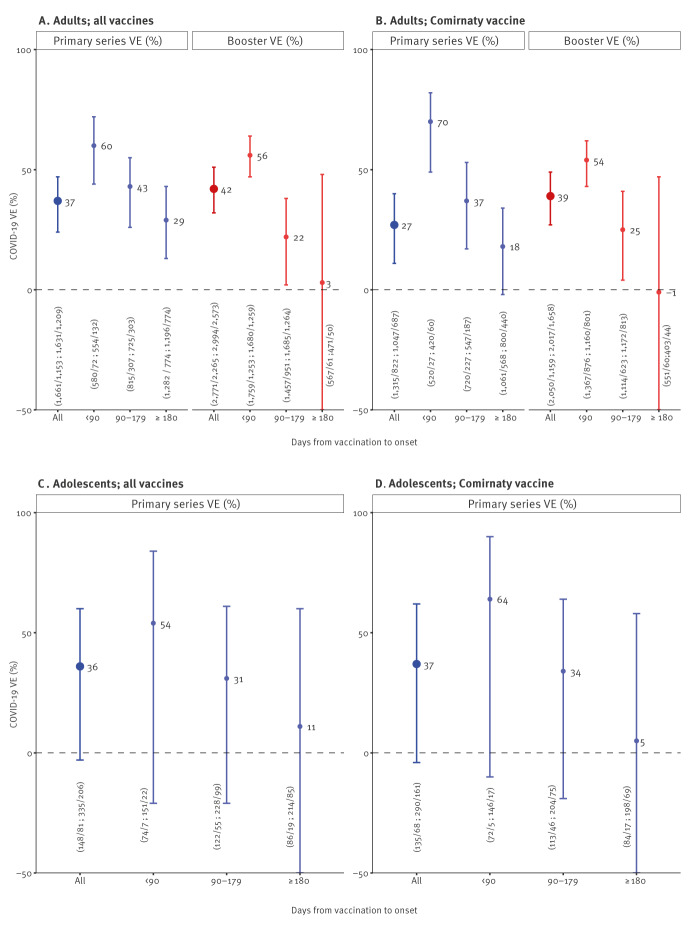
COVID-19 vaccine effectiveness of primary series and first booster vaccination among adults and adolescents by time since vaccination for all vaccine products and for Comirnaty vaccine only, VEBIS primary care study, EU/EEA, December 2021–June 2022

EEA: European Economic Area; EU: European Union; VE: vaccine effectiveness; VEBIS: Vaccine Effectiveness, Burden and Impact Studies.

All VE analysis results are appended in detail in Supplementary Table S1. Among adults included in PS analyses, 69% (1,153/1,661) of cases and 74% (1,209/1,631) of controls had received full PS vaccination (Table 1). Where details on vaccine brand(s) were known (2,077/2,362), 73% (1,509/2,077) of vaccinated patients received two doses of Comirnaty, 4% (89/2,077) one dose of Comirnaty and one dose of another brand, and 23% (479/2,077) other brand(s). All-product primary series VE was 37% (95% confidence interval (CI): 24–47%) overall, and 60% (95% CI: 44–72%), 43% (95% CI: 26–55%) and 29% (95% CI: 13–43%) < 90 days, 90–179 days and ≥ 180 days after vaccination, respectively (Figure 4). Among adults included in the first booster analyses, 82% (2,265/2,771) of cases and 86% (2,573/2,994) of controls had received one booster dose. Where details on PS vaccine brand(s) were known (4,565/4,838), 70% (3,217/4,565) of patients vaccinated with a booster had received two doses of Comirnaty for their PS, 1% (45/4,565) one dose of Comirnaty and one dose of another brand, and 29% (1,303/4,565) other brand(s). All-product first booster VE was 42% (95% CI: 32–51%) overall, and 56% (95% CI: 47–64%), 22% (95% CI: 2–38%) and 3% (95% CI: −78% to 48%) < 90 days, 90–179 days and ≥ 180 days after vaccination, respectively. Corresponding PS and booster VE estimates for adults who received Comirnaty only as PS vaccination are also displayed in Figure 4.

**Table 1:** Baseline characteristics of adults included in primary series and first booster vaccine effectiveness analyses, and adolescents included in primary series analyses, VEBIS primary care study, EU/EEA, December 2021–June 2022

Characteristics	Adults included in primary series analyses				Adults included in first booster analyses				Adolescents included in primary series analyses			
	SARS-CoV-2 cases (n = 1,661)		Test-negative controls (n = 1,631)		SARS-CoV-2 cases (n = 2,771)		Test-negative controls (n = 2,994)		SARS-CoV-2 cases (n = 148)		Test-negative controls (n = 335)	
	n	%	n	%	n	%	n	%	n	%	n	%
Median age (IQR) in years	42 (32–52)		40 (28–52)		52 (41–65)		56 (40–70)		14 (13–16)		14 (13–16)	
Missing	0		0		0		0		0		0	
Sex^a^												
Female	952	57	954	58	1,624	59	1,734	58	67	45	166	50
Male	709	43	677	42	1,147	41	1,260	42	81	55	169	50
Missing	0		0		0		0		0		0	
Chronic condition^b^												
Presence of chronic condition	317	19	361	22	784	28	1,048	35	19	13	39	12
No chronic condition	1,344	81	1,270	78	1,987	72	1,946	65	129	87	296	88
Missing	0		0		0		0		0		0	
COVID-19 vaccination status												
Unvaccinated	508	31	422	26	506	18	421	14	67	45	128	38
Full primary series (only)	1,153	69	1,209	74	0	0	0	0	81	55	207	62
Full primary series + 1 booster (only)	0	0	0	0	2,265	82	2,573	86	0	0	0	0
Missing	0		0		0		0		0		0	
Brand of COVID-19 vaccine: first dose^c^												
Comirnaty	815	77	638	72	1,474	70	1,385	68	67	89	151	88
Spikevax	131	12	125	14	149	7	169	8	8	11	21	12
Vaxzevria	74	7	90	10	362	17	382	19	0	0	0	0
Jcovden	40	4	37	4	120	6	91	4	0	0	0	0
Missing	93		319		160		546		6		35	
Brand of COVID-19 vaccine: second dose^d^												
Comirnaty	839	80	706	75	1,598	73	1,691	72	68	89	161	88
Spikevax	155	15	178	19	263	12	278	12	8	11	21	12
Vaxzevria	60	6	62	7	341	15	391	17	0	0	0	0
Jcovden	0	0	0	0	1	0	0	0	0	0	0	0
Missing	59		226		62		213		5		25	
Brand of COVID-19 vaccine: third dose^e^												
Comirnaty	NA				1,274	60	1,535	66	NA			
Spikevax					831	39	805	34				
Vaxzevria					1	0	1	0				
Jcovden					0	0	0	0				
Missing					39		141					
Study site												
France	203	12	167	10	337	12	327	11	28	19	59	18
Germany	77	5	129	8	155	6	411	14	23	16	69	21
Hungary	13	1	15	1	16	1	20	1	0	0	0	0
Ireland	201	12	185	11	229	8	381	13	31	21	43	13
Navarre	882	53	470	29	1,709	62	1,202	40	54	36	93	28
The Netherlands	23	1	138	8	29	1	184	6	0	0	0	0
Portugal	19	1	48	3	22	1	75	3	0	0	0	0
Romania	32	2	76	5	30	1	32	1	0	0	0	0
Spain	187	11	383	23	223	8	310	10	12	8	71	21
Sweden	24	1	20	1	21	1	52	2	0	0	0	0
Missing	0		0		0		0		0		0	
Dominant variant												
BA.1 (December 2021–February 2022)	838	50	468	29	757	27	714	24	84	57	112	33
BA.2 (February–June 2022)	510	31	865	53	1,408	51	1,672	56	40	27	161	48
Co-circulation	313	19	298	18	606	22	608	20	24	16	62	19
Influenza case status												
RT-PCR-positive for any influenza virus	4	1	210	23	10	2	206	16	1	2	50	30
RT-PCR-negative for all influenza viruses	469	99	705	77	593	98	1,100	84	42	98	118	70
Missing	1,118		716		2,168		1,688		105		167	
History of SARS-CoV-2 infection												
Previously infected	113	9	237	28	83	4	227	12	6	6	34	19
Not previously infected	1,159	91	614	72	2,170	96	1,603	88	99	94	142	81
Missing	389		780		518		1,164		43		159	

EEA: European Economic Area; EU: European Union; IQR: interquartile range; SARS-CoV-2: severe acute respiratory syndrome coronavirus 2; VEBIS: Vaccine Effectiveness, Burden and Impact Studies.

^a^ Sex was collected as a binary variable.

^b^ The presence of chronic condition is defined as reporting at least one of the following conditions: diabetes, immunodeficiency, lung disease, heart disease.

^c^ Percentages are calculated among patients who received at least one vaccine dose.

^d^ Percentages are calculated among patients who received at least two vaccine doses.

^e^ Percentages are calculated among patients who received at least three vaccine doses.

The series of four numbers in vertical text direction provides the number of cases/number of vaccinated cases;number of controls/number of vaccinated controls for each population and time interval. Whiskers indicate 95% confidence intervals.

Among adolescents included in PS analyses, 55% (81/148) of cases and 62% (207/335) of controls had received full PS vaccination. Where details on vaccine brand(s) were known (258/288), 89% (229/258) of vaccinated adolescents had received two doses of Comirnaty and 11% (29/258) other brand(s). Primary series VE was 36% (95% CI: −3 to 60%) overall and 54% (95% CI: −21 to 84%), 31% (95% CI: −21 to 61%) and 11% (95% CI: −94 to 60%) < 90 days, 90–179 days and ≥ 180 days after vaccination, respectively. Corresponding VE estimates for adolescents who received Comirnaty as PS vaccination are also presented in Figure 4.

Among adults aged < 50 years, PS VE was 26% (95% CI: 8–41%) overall and 54% (95% CI: 30–70%), 31% (95% CI: 7–49%) and 16% (95% CI: −8 to 35%) < 90 days, 90–179 days and ≥ 180 days after vaccination, respectively; for the detailed results by subgroups we refer to Supplementary Table S1. In this population, first booster VE was 26% (95% CI: 7–41%) overall and 37% (95% CI: 19–51%) and −34% (95% CI: −84 to 2%) < 90 days and 90–179 days after vaccination, respectively. Sample size was too low to estimate booster VE ≥ 180 days after vaccination in this group.

Among adults aged ≥ 50 years, PS VE was 56% (95% CI: 39–69%) overall and 70% (95% CI: 44–85%), 72% (95% CI: 53–84%) and 50% (95% CI: 28–66%) < 90 days, 90–179 days and ≥ 180 days after vaccination, respectively. In this age group, first booster VE was 59% (95% CI: 46–69%) overall and 73% (95% CI: 63–81%), 48% (95% CI: 28–63%) and 28% (95% CI: −68 to 69%) < 90 days, 90–179 days and ≥ 180 days after vaccination, respectively. The VE estimates stratified by chronic condition status are appended in Supplementary Table S1.

### Results for small sample sizes

Results of Firth’s penalised regression analyses are available in Supplementary Table S2. Of estimates suspected to suffer from small sample size bias, only first booster VE among adults living with a chronic condition had a ≥ 10% difference (at exactly 10%) between per protocol regression and Firth’s penalised regression.

### Sensitivity analyses

Among adults recruited in sites collecting information on influenza case status, overall PS VE was 44% (95% CI: 26–58%) when including influenza-positive controls and 42% (95% CI: 23–56%) when excluding them (Table 2). In this population, the overall first booster VE was 48% (95% CI: 32–60%) when including influenza-positive controls and 49% (95% CI: 33–61%) when excluding them. Among adults recruited in all sites but NA, overall PS VE was 45% (95% CI: 31–56%) and first booster VE was 51% (95% CI: 40–60%) (Table 2).

**Table 2:** Sensitivity and secondary analyses of COVID-19 vaccine effectiveness among adults and adolescents, VEBIS primary care study, EU/EEA, December 2021–June 2022

Population	Regression models	VE analysis	Adjusted VE (95% CI)	n	Vaccinated cases	Unvaccinated cases	Vaccinated controls	Unvaccinated controls
Sensitivity analyses								
Adults from sites collecting information on influenza case status	Per protocol^a^	Primary series	44 (26–58)	1,388	320	153	683	232
		First booster	48 (32–60)	1,940	451	152	1,105	232
Adults from sites collecting information on influenza case status, excluding influenza-positive controls	Per protocol	Primary series	42 (23–56)	1,178	320	153	528	177
		First booster	49 (33–61)	1,731	451	152	951	177
Adults from all sites but Navarra	Per protocol	Primary series	45 (31–56)	1,940	453	326	816	345
		First booster	51 (40–60)	2,854	737	325	1,447	345
Secondary analyses								
Adults from sites collecting information on previous SARS-CoV-2 infection	Per protocol	Primary series	31 (14–45)	2,123	899	373	632	219
		First booster	30 (13–43)	4,083	1,881	372	1,612	218
	Adding previous SARS-CoV-2 infection to per protocol covariates	Primary series	29 (10–44)	2,123	899	373	632	219
		First booster	38 (23–51)	4,083	1,881	372	1,612	218
Adults reporting a previous SARS-CoV-2 infection (in sites collecting this information)	Per protocol	Primary series	29 (−28 to 60)	350	78	35	176	61
		First booster	57 (11–80)	310	48	35	166	61
Adults reporting no previous SARS-CoV-2 infection (in sites collecting this information)	Per protocol	Primary series	27 (6–44)	1,773	821	338	456	158
		First booster	38 (22–51)	3,773	1,833	337	1,446	157
Adults: unvaccinated, previously infected^b^ vs unvaccinated, never infected	Per protocol	Primary series	64 (41–78)	592	35	338	61	158
		First booster	64 (40–79)	590	35	337	61	157
Adults: vaccinated, never infected vs unvaccinated, never infected	Per protocol	Primary series	27 (6–44)	1,773	821	338	456	158
		First booster	38 (22–51)	3,773	1,833	337	1,446	157
Adults: vaccinated, previously infected vs unvaccinated, never infected	Per protocol	Primary series	75 (65–83)	750	78	338	176	158
		First booster	87 (80–92)	708	48	337	166	157

CI: confidence interval; EEA: European Economic Area; EU: European Union; SARS-CoV-2: severe acute respiratory syndrome coronavirus 2; VE: vaccine effectiveness; VEBIS: Vaccine Effectiveness, Burden and Impact Studies.

^a^ The per protocol model is a multivariable logistic regression model adjusted for study site, date of symptom onset, age, sex and the presence of chronic condition.

^b^ When comparing the odds of disease between unvaccinated, never infected patients and unvaccinated, previously infected patients, the measure of effect is the protection conferred by infection rather than vaccine effectiveness.

### Secondary analyses

Among adults recruited in sites collecting information on previous SARS-CoV-2 infection, overall PS VE was 29% (95% CI: −28 to 60%) among adults reporting a previous infection and 27% (95% CI: 6–44%) among those reporting no previous infection (Table 2). First booster VE was 57% (95% CI: 11–80%) among patients reporting a previous infection and 38% (95% CI: 22–51%) among those reporting no previous infection. In the same sites, overall PS VE was 31% (95% CI: 14–45%) when using per protocol modelling and 29% (95% CI: 10–44%) when adding previous SARS-CoV-2 infection to per protocol covariates. The overall first booster VE was 30% (95% CI: 13–43%) per protocol and 38% (95% CI: 23–51%) when including adjustment for previous SARS-CoV-2 infection.

Using as a reference the unvaccinated, never infected adult part of the population selected for PS analyses, the protection conferred by previous infection alone was 64% (95% CI: 41–78%), the protection conferred by PS vaccination alone was 27% (95% CI: 6–44%), and the protection conferred by previous infection plus PS vaccination was 75% (95% CI: 65–83%) (Table 2). Using the same reference group in the population selected for first booster analyses, the protection conferred by previous infection alone was 64% (95% CI: 40–79%), the protection conferred by booster vaccination alone was 38% (95% CI: 22–51%), and the protection conferred by previous infection plus booster vaccination was 87% (95% CI: 80–92%).

## Discussion

COVID-19 VE against symptomatic infection with BA.1/BA.2 ranged from 37% to 42% for overall PS and first booster VE in this VEBIS primary care study among adults and adolescents. The majority of patients had received Comirnaty as PS vaccination, and restricting analyses to these patients yielded similar estimates. Despite some overlap in 95% CIs, all analyses suggested a decline in VE by time since vaccination, with lower or no effect of vaccines after 6 months. This decline was similar for PS and first booster vaccination, with lower booster VE point estimates in some instances. Among adults, PS and first booster VE were similar within 3 months of vaccination, suggesting that the protection provided early after vaccination may be independent of the number of doses received. The VE estimates were higher among older adults. Despite limited sample size, PS VE among adolescents was similar to that among all adults, with higher point estimates among adults for certain time-since-vaccination intervals.

In a meta-analysis published in January 2023, pooled PS COVID-19 VE was 37% at < 3 months, 11% at 3–6 months, and −4% at ≥ 6 months post vaccination, respectively [15]. First booster VE was 57% at < 3 months and 33% at 3–6 months post vaccination. Data were sparse for longer follow-up periods, and the meta-analysis only included two national European studies. Our estimates, using pooled data from 10 European countries up to and over 6 months post-booster vaccination, thereby contribute to the evidence base for COVID-19 vaccination policies in the EU/EAA.

We also leveraged these data to investigate potential bias in VE in primary care studies using the TND. For both PS and first booster VE, our estimates varied by ≤ 2% when excluding influenza-positive, SARS-CoV-2-negative controls. It seems unlikely that COVID-19 VE was biased due to a correlation between influenza vaccination and COVID-19 vaccination in our study, at least in the BA.1/BA.2 circulation period.

We also explored potential confounding and/or effect modification by previous SARS-CoV-2 infection. There was no strong evidence of a difference between estimates obtained with and without adjusting for previous SARS-CoV-2 infection, suggesting a limited risk of confounding. Due to low sample size, we could not draw definite conclusions about potential effect modification by previous SARS-CoV-2 infection. Point estimates were similar among people with and without a history of SARS-CoV-2 infection for PS vaccination. The point estimates of first booster VE were higher among patients with a previous infection, but sample size was low for this group (n = 310), and one CI fully encompassed the other. Nevertheless, VE estimates among patients with and without previous infection show that vaccines can provide further protection against subsequent infections in both groups of patients. When comparing the protection conferred by different combinations of previous infection and vaccination, point estimates were highest among patients who had been infected and vaccinated, in line with recent evidence [23,24]. In these analyses, most of the protection seemed attributable to the immunity generated by previous infection, with lower protection induced by PS or booster dose vaccination alone.

Precision was limited in our study and some of our bias analysis results should be investigated by further work. Another limitation is that information on previous SARS-CoV-2 infection was self-reported in most sites and relied on patients having undergone testing. Compared with more objective measurements (e.g. regular serological testing of infection-induced antibodies), self-reported infection can be subject to measurement error and recall bias and depends on testing behaviour. Furthermore, not all study sites provided information on previous infection, and we missed parameters such as the date and number of previous infections.

Although our VE estimates are consistent with those of the meta-analysis published by Mohammed et al. [15] and other studies of VE against Omicron lineages [25-27], an apparent VE decline could be caused by the depletion of susceptible individuals at different rates among vaccinated and unvaccinated people in a context of high incidence [28].

Unexpectedly, VE estimates were higher among older adults (aged ≥ 50 years) in our study. One plausible explanation is a difference in behaviour and previous exposure to SARS-CoV-2. Younger adults may have greater contact patterns than older adults [29]. If we assume that the vaccine offers protection against infection, then we would have a higher proportion of recently infected patients in the unvaccinated younger age group than in the unvaccinated older age group. This could artificially lower apparent VE estimates among younger adults [30]. Unfortunately, our data did not allow us to fully explore these hypotheses.

Other studies have reported similar VE and temporal trends among adolescents [31]. In a national study conducted in the United Kingdom, Powell et al. estimated PS VE against symptomatic infection with BA.1/BA.2 among adolescents [11]. Their estimates varied widely by patients’ history of SARS-CoV-2 infection and declined with time since vaccination.

## Conclusions

Our results indicate that timing of potential booster vaccination may be critical for priority populations, for example to reduce pressure on healthcare services in the context of high SARS-CoV-2 incidence. These findings also indicate that estimating VE by time since last dose among all who have received (at least) PS vaccination may be sufficient, rather than estimating VE by number of doses. Collecting comprehensive data on history of SARS-CoV-2 infection would help disentangle vaccine- and infection-induced immunity and better estimate age-specific VE.

## Supplementary Data

229000 Supplement
