# Supplementary material for: COVID-19 vaccine effectiveness against symptomatic infection with SARS-CoV-2 BA.1/BA.2 lineages among adults and adolescents in a multicentre primary care study, Europe, December 2021 to June 2022
Source: Euro Surveill. 2024 Mar 28;29(13):2300403. doi: 10.2807/1560-7917.ES.2024.29.13.2300403 (PMC10979526; doi:10.2807/1560-7917.ES.2024.29.13.2300403)
Supplement: Supplement [file 23-00403_DELAUNAY_Supplement.pdf]

COVID-19 vaccine effectiveness against symptomatic infection with SARS-CoV-2 BA.1/BA.2 lineages among adults and adolescents in a multicentre primary care study in Europe

Supplementary methods and results

This supplementary material is hosted by *Eurosurveillance* as supporting information alongside the article "COVID-19 vaccine effectiveness against symptomatic infection with SARS-CoV-2 BA.1/BA.2 lineages among adults and adolescents in a multicentre primary care study in Europe", on behalf of the authors, who remain responsible for the accuracy and appropriateness of the content. The same standards for ethics, copyright, attributions and permissions as for the article apply. Supplements are not edited by *Eurosurveillance* and the journal is not responsible for the maintenance of any links or email addresses provided therein.

Supplementary methods

Site-specific variations in case definition for study inclusion

In each site, participating physicians swabbed all or a systematic sample of patients consulting with symptoms meeting either the EU acute respiratory infection (ARI) case definition (sudden onset of symptoms AND at least one the following four respiratory symptoms: cough, sore throat, shortness of breath or coryza AND a clinician's judgement that the illness is due to an infection) or COVID-19-like symptoms (addition of anosmia/ageusia to the ARI case definition).

There were minor variations across sites in the case definition used to determine the eligibility of patients to participate in the study. FR used the following case definition: sudden onset of fever (or feverishness) and respiratory symptoms. Case definitions are also subject to change, and the ones described above are relevant to the study period covered by this analysis.

Once patients were deemed eligible to participate, the approach used to select participants to include in the study varied across sites. HR, HU, IE, NA and RO selected all patients meeting the chosen case definition. In DE and FR, age-stratified sampling was performed. DE selected the first two patients in the age groups 15 to 34 years and 35 to 59 years, and the first six patients among those above 60 years old. In FR, each week, the first patient under 65 years old and the first patient 65 years old or above were selected into the study. In PT, physicians selected the first four to five patients presenting to them on a given week day (the week day changed from one week to another). In ES, the first two to five patients seen by the physician on a given week were selected. SE proceeded similarly to ES (selection of the first five patients).

Functional forms used to model variables in per protocol logistic regression models

We used logistic regression models to estimate vaccine effectiveness as per the following formula:

$$Vaccine\ effectiveness = 1 - \frac{odds\ of\ vaccination\ in\ cases}{odds\ of\ vaccination\ in\ controls} * 100$$

All logistic regression models were adjusted for the following a priori confounders:

- Study site;
- Date of symptom onset;
- Age;
- Sex;
- Presence of at least one chronic condition including diabetes, immunodeficiency, lung disease, and heart disease (except in analyses stratified by chronic condition status).

Study site was modelled as a categorical variable, and sex and presence of chronic condition as binary variables. Date of symptom onset was modelled successively as a categorical variable (with one category per week) and with restricted cubic splines with

three, four, and five knots. Age was modelled successively as a continuous variable, as a categorical variable (with 10-year age categories), and with restricted cubic splines with three, four, and five knots.

For each analysis, we ran a total of 27 models to determine the optimal functional form of the age and symptom onset variables. We used the Akaike Information Criterion (AIC) to choose the best fitting model, and we considered the magnitude of regression coefficients and their standard errors, in case inflated coefficients suggested unstable modelling results.

Supplementary results

Table S1. COVID-19 vaccine effectiveness estimates for primary series and first booster vaccination among adults and adolescents, VEBIS primary care study, EU/EEA, December 2021–June 2022.

VE analysis	Population	Regression models	Time since vaccination (in days)	Adjusted VE (95 % CI)	N	Vaccinated cases	Unvaccinated cases	Vaccinated controls	Unvaccinated controls
**Main analyses**									
Primary series VE									
Primary series	All adults	Per protocol[a]	Any	37 (24; 47)	3,292	1,153	508	1,209	422
			<90	60 (44; 72)	1,134	72	508	132	422
			90–179	43 (26; 55)	1,540	307	508	303	422
			≥180	29 (13; 43)	2,478	774	508	774	422
Primary series	Adults who received Comirnaty as primary series	Per protocol	Any	27 (11; 40)	2,362	822	493	687	360
			<90	70 (49; 82)	940	27	493	60	360
			90–179	37 (17; 53)	1,267	227	493	187	360
			≥180	18 (-2; 34)	1,861	568	493	440	360
Primary series	Adults aged <50 years	Per protocol	Any	26 (8; 41)	2,300	862	311	821	306
			<90	54 (30; 70)	758	48	311	93	306
			90–179	31 (7; 49)	1,119	270	311	232	306
			≥180	16 (-8; 35)	1,657	544	311	496	306
Primary series	Adults aged ≥50 years	Per protocol	Any	56 (39; 69)	992	291	197	388	116
			<90	70 (44; 85)	376	24	197	39	116
			90–179	72 (53; 84)	421	37	197	71	116
			≥180	50 (28; 66)	821	230	197	278	116
Primary series	Adults with no chronic condition[b]	Per protocol (no adjustment for chronic condition)	Any	38 (25; 50)	2,614	921	423	920	350

VE analysis	Population	Regression models	Time since vaccination (in days)	Adjusted VE (95 % CI)	N	Vaccinated cases	Unvaccinated cases	Vaccinated controls	Unvaccinated controls
			<90	58 (38; 72)	939	61	423	105	350
			90–179	43 (26; 57)	1,285	261	423	251	350
			≥180	32 (15; 46)	1,936	599	423	564	350
Primary series	Adults with a chronic condition	Per protocol	Any	26 (-13; 52)	678	232	85	289	72
			<90	70 (30; 88)	195	11	85	27	72
			90–179	32 (-33; 65)	255	46	85	52	72
			≥180	16 (-36; 48)	542	175	85	210	72
Primary series	All adolescents	Per protocol	Any	36 (-3; 60)	483	81	67	206	129
			<90	54 (-21; 84)	225	7	67	22	129
			90–179	31 (-21; 61)	350	55	67	99	129
			≥180	11 (-94; 60)	300	19	67	85	129
Primary series	Adolescents who received Comirnaty as primary series	Per protocol	Any	37 (-4; 62)	425	68	67	161	129
			<90	64 (-10; 90)	218	5	67	17	129
			90–179	34 (-19; 64)	317	46	67	75	129
			≥180	5 (-113; 58)	282	17	67	69	129
First booster VE									
First booster	All adults	Per protocol	Any	42 (32; 51)	5,765	2,265	506	2,573	421
			<90	56 (47; 64)	3,439	1,253	506	1,259	421
			90–179	22 (2; 38)	3,142	951	506	1,264	421
			≥180	3 (-78; 48)	1,038	61	506	50	421
First booster	Adults who received Comirnaty as primary series	Per protocol	Any	39 (27; 49)	4,067	1,559	491	1,658	359
			<90	54 (43; 62)	2,527	876	491	801	359
			90–179	25 (4; 41)	2,286	623	491	813	359

VE analysis	Population	Regression models	Time since vaccination (in days)	Adjusted VE (95 % CI)	N	Vaccinated cases	Unvaccinated cases	Vaccinated controls	Unvaccinated controls
			≥180	-1 (-90; 47)	954	60	491	44	359
First booster	Adults aged <50 years	Per protocol	Any	26 (7; 41)	2,387	911	309	862	305
			<90	37 (19; 51)	1,763	620	309	529	305
			90–179	-34 (-84; 2)	1,232	289	309	329	305
			≥180	NA	620	2	309	4	305
First booster	Adults aged ≥50 years	Per protocol	Any	59 (46; 69)	3,378	1,354	197	1,711	116
			<90	73 (63; 81)	1,676	633	197	730	116
			90–179	48 (28; 63)	1,910	662	197	935	116
			≥180	28 (-68; 69)	418	59	197	46	116
First booster	Adults with no chronic condition	Per protocol	Any	44 (32; 54)	3,933	1,566	421	1,597	349
			<90	57 (46; 66)	2,557	932	421	855	349
			90–179	16 (-9; 35)	2,110	615	421	725	349
			≥180	-3 (-155; 59)	806	19	421	17	349
First booster	Adults with a chronic condition	Per protocol	Any	45 (20; 62)	1,832	699	85	976	72
			<90	56 (33; 72)	882	321	85	404	72
			90–179	42 (9; 64)	1,032	336	85	539	72
			≥180	-57 (-494; 56)	232	42	85	33	72
**Sensitivity analyses**									
Primary series VE									
Primary series	Adults from sites collecting information on influenza case status	Per protocol	Any	44 (26; 58)	1,336	313	143	659	221
Primary series	Adults from sites collecting information on influenza case status,	Per protocol	Any	42 (23; 56)	1,143	313	143	516	171

VE analysis	Population	Regression models	Time since vaccination (in days)	Adjusted VE (95 % CI)	N	Vaccinated cases	Unvaccinated cases	Vaccinated controls	Unvaccinated controls
	excluding influenza-positive controls								
Primary series	Adults from all sites but Navarra	Per protocol	Any	45 (31; 56)	1,940	453	326	816	345
1st booster VE									
First booster	Adults from sites collecting information on influenza case status	Per protocol	Any	48 (32; 60)	1,845	434	142	1,048	221
First booster	Adults from sites collecting information on influenza case status, excluding influenza-positive controls	Per protocol	Any	49 (33; 61)	1,643	434	142	896	171
First booster	Adults from all sites but Navarra	Per protocol	Any	51 (40; 60)	2,854	737	325	1,447	345
**Secondary analyses**									
Primary series VE									
Primary series	Adults reporting a previous SARS-CoV-2 infection (in sites collecting this information)	Per protocol	Any	29 (-28; 60)	350	78	35	176	61
Primary series	Adults reporting no previous SARS-CoV-2 infection (in sites collecting this information)	Per protocol	Any	27 (6; 44)	1,773	821	338	456	158
Primary series	Adults from sites collecting information on previous SARS-CoV-2 infection	Per protocol	Any	31 (14; 45)	2,123	899	373	632	219
Primary series	Adults from sites collecting information on	Adding previous SARS-CoV-2	Any	29 (10; 44)	2,123	899	373	632	219

VE analysis	Population	Regression models	Time since vaccination (in days)	Adjusted VE (95 % CI)	N	Vaccinated cases	Unvaccinated cases	Vaccinated controls	Unvaccinated controls
	previous SARS-CoV-2 infection	infection to per protocol covariates							
Primary series	Adults: unvaccinated, never infected vs. unvaccinated, previously infected[b]	Per protocol	Any	64 (41; 78)	592	35	338	61	158
Primary series	Adults: unvaccinated, never infected vs. vaccinated, never infected	Per protocol	Any	27 (6; 44)	1,773	821	338	456	158
Primary series	Adults: unvaccinated, never infected vs. vaccinated, previously infected	Per protocol	Any	75 (65; 83)	750	78	338	176	158
First booster VE									
First booster	Adults reporting a previous SARS-CoV-2 infection (in sites collecting this information)	Per protocol	Any	57 (11; 80)	310	48	35	166	61
First booster	Adults reporting no previous SARS-CoV-2 infection (in sites collecting this information)	Per protocol	Any	38 (22; 51)	3,773	1,833	337	1,446	157
First booster	Adults from sites collecting information on previous SARS-CoV-2 infection	Per protocol	Any	30 (13; 43)	4,083	1,881	372	1,612	218
First booster	Adults from sites collecting information on previous SARS-CoV-2 infection	Adding previous SARS-CoV-2 infection to per protocol covariates	Any	38 (23; 51)	4,083	1,881	372	1,612	218
First booster	Adults: unvaccinated, never infected vs.	Per protocol	Any	64 (40; 79)	590	35	337	61	157

VE analysis	Population	Regression models	Time since vaccination (in days)	Adjusted VE (95 % CI)	N	Vaccinated cases	Unvaccinated cases	Vaccinated controls	Unvaccinated controls
	unvaccinated, previously infected[b]								
First booster	Adults: unvaccinated, never infected vs. vaccinated, never infected	Per protocol	Any	38 (22; 51)	3,773	1,833	337	1,446	157
First booster	Adults: unvaccinated, never infected vs. vaccinated, previously infected	Per protocol	Any	87 (80; 92)	708	48	337	166	157

CI: confidence interval; VE: vaccine effectiveness.

[a]The per protocol model is a multivariable logistic regression model adjusted for study site, date of symptom onset, age, sex, and the presence of chronic condition.

[b]When comparing the odds of disease between unvaccinated, never infected patients and unvaccinated, previously infected patients, the measure of effect is the protection conferred by infection rather than vaccine effectiveness.

Table S2. COVID-19 vaccine effectiveness among adults and adolescents, using 1) per protocol modelling and 2) Firth's penalised regression (1)**, VEBIS primary care study, EU/EEA, December 2021–June 2022.**

VE analysis	Population	Regression models	Time since vaccination (in days)	Adjusted VE (95 % CI)	N	Vaccinated cases	Unvaccinated cases	Vaccinated controls	Unvaccinated controls
Primary series	Adults aged ≥50 years	Per protocol[a]	<90	70 (44; 85)	376	24	197	39	116
Primary series	Adults aged ≥50 years	Penalised regression	<90	69 (42; 84)	376	24	197	39	116
Primary series	Adults with a chronic condition	Per protocol	<90	70 (30; 88)	195	11	85	27	72
Primary series	Adults with a chronic condition	Penalised regression	<90	66 (26; 86)	195	11	85	27	72
Primary series	Adults with a chronic condition	Per protocol	90-179	32 (-33; 65)	255	46	85	52	72
Primary series	Adults with a chronic condition	Penalised regression	90-179	30 (-32; 64)	255	46	85	52	72
First booster	Adults with a chronic condition	Per protocol	≥180	-57 (-494; 56)	232	42	85	33	72
First booster	Adults with a chronic condition	Penalised regression	≥180	-47 (-423; 56)	232	42	85	33	72
Primary series	All adolescents	Per protocol	<90	54 (-21; 84)	225	7	67	22	129
Primary series	All adolescents	Penalised regression	<90	50 (-25; 82)	225	7	67	22	129
Primary series	All adolescents	Per protocol	≥180	11 (-94; 60)	300	19	67	85	129
Primary series	All adolescents	Penalised regression	≥180	10 (-93; 58)	300	19	67	85	129
Primary series	Adolescents who received Comirnaty as primary series	Per protocol	<90	64 (-10; 90)	218	5	67	17	129
Primary series	Adolescents who received Comirnaty as primary series	Penalised regression	<90	60 (-15; 88)	218	5	67	17	129

VE analysis	Population	Regression models	Time since vaccination (in days)	Adjusted VE (95 % CI)	N	Vaccinated cases	Unvaccinated cases	Vaccinated controls	Unvaccinated controls
Primary series	Adolescents who received Comirnaty as primary series	Per protocol	≥180	5 (-113; 58)	282	17	67	69	129
Primary series	Adolescents who received Comirnaty as primary series	Penalised regression	≥180	4 (-110; 57)	282	17	67	69	129

CI: confidence interval; VE: vaccine effectiveness.

[a] The per protocol model is a multivariable logistic regression model adjusted for study site, date of symptom onset, age, sex, and the presence of chronic condition.

References

1. Firth D. Bias reduction of maximum likelihood estimates. Biometrika. 1993;80(1):27–38.
